# Macao's COVID-19 responses: From virus elimination success to vaccination rollout challenges

**DOI:** 10.1016/j.lanwpc.2021.100169

**Published:** 2021-06-09

**Authors:** Glenn McCartney, Jose Pinto

**Affiliations:** 1 Associate Dean (Teaching & Learning), Associate Professor in International Integrated Resort Management. Faculty of Business Administration, Avenida da Universidade, Taipa, University of Macau; 2 PhD student, Faculty of Business Administration, Avenida da Universidade, Taipa, University of Macau

**Keywords:** Macao, COVID-19 eradication, vaccination rollout

Macao's ability to quickly identify, contain, and eradicate COVID-19 in the city offers an important perspective on healthcare delivery, public security, community solidarity, and city governance. While this commentary features Macao's COVID-19 response success and current vaccination rollout, it also brings to the fore the growing disparity and inequity in Asia of securing vaccine providers and vaccines, and a judicious and timely rollout schedule. Neighbouring jurisdictions such as the Philippines have secured vaccines through the COVAX facility for less than 1% of the population, with predictions of 2023 to inoculate the population [1]. Access to global vaccine supplies and the emergence of new COVID-19 variants have created a public health strain on Asian jurisdictions, now challenged to hasten community inoculation [2]. These concerns to speed vaccination rollout prompted Vietnam to introduce the domestically produced Nanocovax vaccine by 2022 [3]. While not a vaccine supply concern, Macao's next COVID-19 response challenge is to ramp-up inoculation.

Paradoxically, Macao's success in COVID-19 eradication by mid-July 2020 and returning to everyday community life - with required mandates - created a low city-population vaccination rate. Residents feel less risk of infection, adopt a ‘wait-and-see’ stance, and seek assurances that the vaccines are safe. The city has 600,425 doses of vaccine (200,000 Sinopharm inactivated vaccine doses and 400,425 BioNTech mRNA doses), previously securing and purchasing a total of 1.4 billion doses of vaccine through the WHO Covax scheme - sufficient for the entire population [4]. The authorities have appealed for herd immunity, with most of the population inoculated a key government goal [5]. Only 35,000 were vaccinated by mid-March, despite the government health bureau efforts to communicate vaccination information across the city [6].

The three-phase free-of-charge voluntary vaccination process in Macao with a user-friendly online and mobile booking platform and multiple vaccination sites started on Feb 9. Macao's vaccination targets are similar to other low-risk jurisdictions, and therefore initially excluded the elderly. Phase 1 included frontline workers, school staff, and those employed in the city's economically important tourism and casino sectors. Sinopharm was only available, with criteria tentatively set for those between 18 and 60 years. The National Medical Products Administration of China instructed that Sinopharm could only be used on those over 60 with good health and had a high risk of exposure [7]. BioNTech was included in phase 2 rollout (Feb 22) for all Macao residents over 16 years. In phase 3 (Mar 10), the complimentary vaccines were available to the city's non-resident workers, who make up almost half of the 400,000 workforce – and mainly from Mainland China, Philippines, Vietnam, Hong Kong, and Indonesia [6].

As one of the world's most densely populated cities at 20,400 people per km^2^, and a population of 680,000: Macao received 39.4 million visitors (70% from Mainland China) in 2019 (1/58 local-to-visitor ratio) [8]. Under such conditions, rapid COVID-19 containment is crucial to prevent community spread. Macao eliminated COVID-19 in July 2020, discharging the 46^th^ case from the hospital. Between 22^nd^ to Jan 27, Macao hospitalized the first seven cases of COVID-19, all visitors from Wuhan [5], although future cases included Macao residents and non-resident workers. Macao's Pandemic Efficiency Index (PEI) is zero, with no COVID-19 fatalities. The PEI is a metric to illustrate success in containing and managing COVID-19 [9].

The Macao authorities implemented a ‘closed loop’ system to detect and prevent community spread of COVID-19. Again, the process's success was highlighted when an asymptomatic resident entering Macao tested positive to the required two nucleic-acid amplification tests (NAAT). Macao's 47^th^ COVID-19 case was on a flight returning with Macao residents in January 2021, travelling from high-risk countries [4]. Macao has cautiously reopened, establishing a cross-border travel border with China on Sep 23, with over 2.2 million Mainland Chinese visitors (92% of total visitation) from October 2020 to January 2021. Travellers must provide a negative COVID-19 test before departure to Macao, including health-code system phone app registration. This travel corridor success is notable, given global attempts to establish similarly.

The authorities have gauged reopening responses to changes in the COVID-19 situation. After 400 COVID-19 cases were reported in Hebei and other sporadic cases in China, the Macao authorities urged both citizens and non-resident workers to remain in Macao for the Chinese New Year in February 2021 [10]. Chinese New Year celebrations were abruptly cancelled in 2020 as the first cases of COVID-19 emerged in Macao. Downgrading the remaining two cities in China from ‘medium risk’ to ‘low risk’ in late February 2021 meant all travellers from China could now enter quarantine-free, wavering the 14-days mandated hotel quarantined although still requiring a negative COVID-19 certificate [4]. The public health mandates and key take-aways from lessons learnt on Macao's COVID-19 eradication, transmission-reduction tactics, and monitoring success, provided the impetus for the city's recovery and reopening. The interventions and containment measures in place for over a year of COVID-19 NAAT testing, hotel quarantining, health code app, and mask mandate are well documented [5]. In tandem with COVID-19 control measures, the city's politico-administrative state has been accredited for the city's ability to react swiftly to implement these mandates [8]. From the outset, a city-wide communication programme sought community cooperation to adhere to public health mandates. Due to the mix nationality workforce, communication was in English and the official Chinese and Portuguese languages. Research has shown a high level of satisfaction and trust by most Macao residents towards handling the pandemic and disseminating information [11]. However, an outcome has meant reduced risk perceptions and a subsequence low vaccination rate.

In addition to safety perceptions limiting intent to get vaccinated, the Macao health authorities have had to communicate and dispel several rumours and false narratives on the safety and efficacy of the vaccines - namely that immunization: contains a microchip injected to control the human brain and body; will change one's genes; be the cause of a positive NAAT; cause Bell's palsy; and could lead to death [6]. With no severe side effects so far, the Macao authorities have remained vigilant to counteract any negative vaccination news within the city or externally and underscore its two vaccines’ safety and efficacy.

Community economic well-being remained a vital aim of the authorities throughout COVID-19. As one of the wealthiest cities per capita globally, the authorities immediately provided US$4.8 billion in financial assistance packages and cash aid for local individuals and small businesses [12]. The financial assistance continued, instilling confidence within the community. Plausible scenarios in which inoculation numbers swell may include: heightened perceived risk from COVID-19 infections; incentivization (e.g. time off work, financial); far-reaching benefits (e.g. travel requirement); or becoming mandated for certain workers - throughout 2020, the authorities sent administrative orders to the casino and hospitality industry, one requiring that all casino staff and customers must have COVID-19 testing [13]. An area to examine further would be the vaccination uptake with phase 3 now underway, including an investigation of non-resident to resident vaccination rates, motives, and risk perceptions.

While maintaining a city safe from COVID-19, Macao is now challenged to quicken its community immunity goal, given the onset of multiple COVID-19 variants in Asia. Macao is economically dependent on tourism, and with this, the continuous opening of borders – the travel bubble has ensured some visitation. Still, it is limiting, and more is needed to bring the industry to profit (12). A vaccinated population, many as frontline workers, would enhance visitor confidence, trust, and safety sentiments. Macao presents a notable case study on lessons learnt, with vaccination rollout throughout Asia and borders reopening.

## Author contributions

GM designed the commentary and takes responsibility for the integrity and accuracy of the information. JP provided review and comments. All authors critically revised the manuscript and approved the version to be published.

## Declaration of Competing Interest

None of the authors have any competing interests to report.
